# ADAMTS-10 and -6 differentially regulate cell-cell junctions and focal adhesions

**DOI:** 10.1038/srep35956

**Published:** 2016-10-25

**Authors:** Stuart A. Cain, Ewa J. Mularczyk, Mukti Singh, Teresa Massam-Wu, Cay M. Kielty

**Affiliations:** 1 Wellcome Trust Centre for Cell-Matrix Research, Faculty of Biology, Medicine and Health, University of Manchester, Manchester M13 9PT, United Kingdom

## Abstract

ADAMTS10 and ADAMTS6 are homologous metalloproteinases with ill-defined roles. ADAMTS10 mutations cause Weill-Marchesani syndrome (WMS), implicating it in fibrillin microfibril biology since some fibrillin-1 mutations also cause WMS. However little is known about ADAMTS6 function. ADAMTS10 is resistant to furin cleavage, however we show that ADAMTS6 is effectively processed and active. Using siRNA, over-expression and mutagenesis, it was found ADAMTS6 inhibits and ADAMTS10 is required for focal adhesions, epithelial cell-cell junction formation, and microfibril deposition. Either knockdown of ADAMTS6, or disruption of its furin processing or catalytic sites restores focal adhesions, implicating its enzyme activity acts on targets in the focal adhesion complex. In ADAMTS10-depleted cultures, expression of syndecan-4 rescues focal adhesions and cell-cell junctions. Recombinant C-termini of ADAMTS10 and ADAMTS6, both of which induce focal adhesions, bind heparin and syndecan-4. However, cells overexpressing full-length ADAMTS6 lack heparan sulphate and focal adhesions, whilst depletion of ADAMTS6 induces a prominent glycocalyx. Thus ADAMTS10 and ADAMTS6 oppositely affect heparan sulphate-rich interfaces including focal adhesions. We previously showed that microfibril deposition requires fibronectin-induced focal adhesions, and cell-cell junctions in epithelial cultures. Here we reveal that ADAMTS6 causes a reduction in heparan sulphate-rich interfaces, and its expression is regulated by ADAMTS10.

ADAMTS6 and ADAMTS10 are closely-related members of the ADAMTS (A Disintegrin And Metalloproteinase with ThromboSpondin Motifs) family, with ill-defined roles. Recessive mutations in ADAMTS10 cause Weill-Marchesani syndrome (WMS)^1^,^2^ associated with short stature, thickened skin and cornea, fibrotic cardiac valves and lens defects. WMS is also caused by certain dominant mutations in fibrillin-1, indicating an unexpected functional relationship between ADAMTS10 and fibrillin microfibrils.

ADAMTS enzymes have an N-terminal catalytic domain and C-terminal region containing thrombospondin type 1-like (TSR) repeats. Secreted as zymogens, most are activated pericellularly upon removal of N-propeptides by furin; however, ADAMTS10 is normally resistant to furin cleavage^3^,^4^.

The functional link between ADAMTS10 and fibrillin-1 is unclear. Fibrillin is the main component of microfibrils that are indispensable components of elastic fibres^5^ that transmit force^6^ and regulate bioavailability of transforming growth factor-beta (TGFβ) family members^7^. Whilst most mutations in fibrillin-1 cause Marfan syndrome^8^, a few cause stiff skin syndrome^9^, WMS^10^,^11^,^12^ or acromicric and geleophysic dysplasias (AD, GD)^2^,^13^. Fibrillin-1 WMS mouse showed a thickened dermis, which when examined by electron microscopy contained abundant disordered microfibrils^12^. ADAMTS10 colocalises with microfibrils in superficial dermis and fibroblast cultures, and in zonules, and can interact with fibrillin-1^3^.

Heparan sulphate (HS) plays an important role in microfibril deposition, which is blocked by exogenous heparin^14^,^15^. Fibrillin-1 binds HS at multiple sites and HS regulates its multimerization^16^,^17^, whilst fibrillin-1 multimers enhance HS interactions^18^. We showed that fibrillin-1 TB5 domain (site of most WMS, AD and GD mutations) binds HS and can induce focal adhesions^19^, and that all tested mutations disrupted this interaction^10^. Microfibril deposition involves focal adhesion-inducing fibronectin (FN), and focal adhesion receptors syndecan-4 and α5β1 integrin^20^,^21^.

We compared ADAMTS10 with its homologue ADAMTS6, in order to gain insights into how these molecules affect focal adhesions, cell-cell junctions and microfibrils. We found that ADAMTS6 disrupts the HS-rich cell interfaces, such as focal adhesions, implicated in microfibril deposition. Whereas ADAMTS10 is needed to support, HS-rich cell interfaces, possibly by regulation of ADAMTS6. Syndecan-4 and other proteoglycans on the cell surface, along with glycoproteins form a carbohydrate-rich layer termed the glycocalyx. Computational modelling suggest that the glycocalyx is a potent regulator of integrin clustering along with the interaction with the ECM^22^. We also show that glycocalyx on the surface of ARPE-19A cells was dramatically altered with the depletion of ADAMTS6 and ADAMTS10, suggesting a possible mechanism for the disruption of focal adhesions and cell-cell interactions.

## Results

### ADAMTS10 supports but ADAMTS6 inhibits focal adhesions

Due to the importance of focal adhesions in microfibril deposition^20^, we explored whether ADAMTS10 and ADAMTS6 affect focal adhesions in human pigmented retinal epithelial ARPE-19A cells^20^, in murine EpH4 mammary epithelial cells^23^,^24^, and in adherent mesenchymal cultures of human dermal fibroblasts (HDFs).

#### Effects of ADAMTS overexpression on focal adhesions

**Figure 1:**
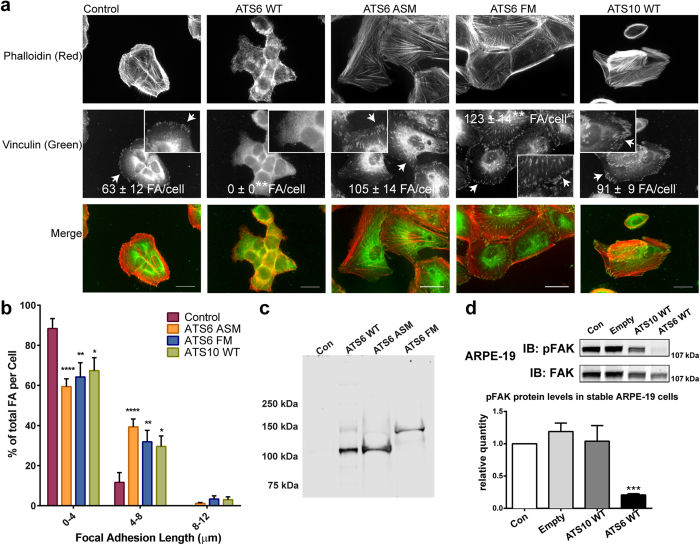
Overexpression of active ADAMTS6 inhibits focal adhesions, but not ADAMTS10. **(a)** Immunofluorescence microscopy of ARPE-19A cells overexpressing ADAMTS6 or ADAMTS10. Focal adhesions were visualised by immuno-staining of vinculin (B/W, green) and phalloidin (B/W, red) on glass coverslips after 3 days. Shown are cells overexpressing ADAMTS6 wild type (ATS6 WT), an ADAMTS6 active site mutant (ATS6 ASM), an ADAMTS6 furin cleavage site mutant (ATS6 FM) and cells overexpressing ADAMTS10 wild type (ATS10 WT). Control cells were infected with lentivirus containing an empty vector. ATS6 WT cells had no detectable focal adhesions; however more focal adhesions per cell were detected with ATS6 ASM, ATS6 FM and ATS10 WT. Examples of focal adhesions are indicated by arrows. The number of focal adhesions (FA) per cell and length was calculated by manual annotation using ImageJ (FA number indicated (n = 5 cells)). Images were taken with a 40x objective. Scale bars indicate 50 μm. **(b)** FA lengths were categorised into 3 groups (0–4, 4–8 and 8–12 μm). The graph shows % of the total FA per cell of the 3 length groups. Cells overexpressing ATS6 ASM, ATS6 FM and ATS10 WT had significant increases in longer FA (4–8 μm). (focal adhesion measurements per cell n > 20). **(c)** Western blot of media taken from ARPE-19A cells overexpressing ADAMTS6 variants. ATS FM cannot be cleaved by furin so still contains the pro-domain. **(d)** ARPE-19A cells overexpressing ADAMTS6 (ATS6 WT) had greatly reduced pFAK in ARPE-19A. Western blotting analysis for total FAK and pFAK is shown. Quantification of band intensity is shown as a ratio of the control band intensity (n = 3). The western blot shown is from a representative experiment. Statistical significance for deviation from the control values was calculated using 1-way ANOVA (2-way ANOVA for FA length comparisons) with a Bonferroni’s multiple comparisons test using GraphPad Prism V6. Asterisk indicate P values where *P ≤ 0.05; **P ≤ 0.01; ***P ≤ 0.001; ****P ≤ 0.0001.

We overexpressed full-length human ADAMTS10 or ADAMTS6 in ARPE-19A and EpH4 epithelial cells by lentiviral vector, with green fluorescent protein (GFP) fluorescence-activated cell sorting to exclude non-expressors and the highest expressors. ARPE-19A and EpH4 cells overexpressing ADAMTS6 (ATS6 WT) had no observable focal adhesions (Fig. 1a, Supplementary Fig. 1a). To negate the catalytic activity of ADAMTS6, two mutants were created; the first mutation was in the metalloprotease active site motif (ATS6 ASM) where the peptide sequence was changed from H**E**IGHNFGMNHD to H**A**IGHNFGMNHD. The second mutation was inserted into the furin cleavage site (ATS6 FM), cleavage of the pro-domain is may be needed for activation of the ADAMTS6. Overexpression of ADAMTS6 mutants ATS6 ASM and ATS6 FM both resulted in increases in focal adhesions, compared to control, showing a dominant negative effect (Fig. 1a). Focal adhesion lengths were grouped into 3 categories (0–4, 4–8 and 8–12 nm); both mutants had a significantly greater percentage of focal adhesions of length between 4–8 nm, and also contained longer focal adhesions (8–12 nm) than those seen in the control cells (Fig. 1b). ADAMTS10 overexpressing cells (ATS10 WT) had more prominent focal adhesions in ARPE-19A and EpH4 cells (Fig. 1a, Supplementary Fig. 1a) and a significantly greater percentage of longer focal adhesions than control cells.

Serum-free conditioned media (CM) was taken from control and ADAMTS6 overexpressing cells. Western blotting analysis of the media from the ATS6 FM cells confirmed that ADAMTS6 was unprocessed by furin and had an apparent molecular weight of 154 kDa, compared with 115 kDa for both ATS6 WT and ATS6 ASM (Fig. 1c). When the serum-free conditioned media was added to wild-type ARPE-19A, there was a significant decrease in the amount of focal adhesions induced by the media from ATS6 WT overexpressing cells, but not conditioned media from ATS6 FM or ATS6 ASM (Supplementary Fig. 1b). Focal adhesion length was not significantly altered after the addition of ATS6 WT CM and ATS6 ASM CM, however the addition of ATS6 FM CM resulted in an increase of focal adhesion length (Supplementary Fig. 1c). ATS6 WT overexpressing cells had significantly lower pFAK in both ARPE-19A cells and EpH4 cells (Fig. 1d, Supplementary Fig. 1d).

#### Effects of ADAMTS knock-down on gene expression and focal adhesions

**Figure 2:**
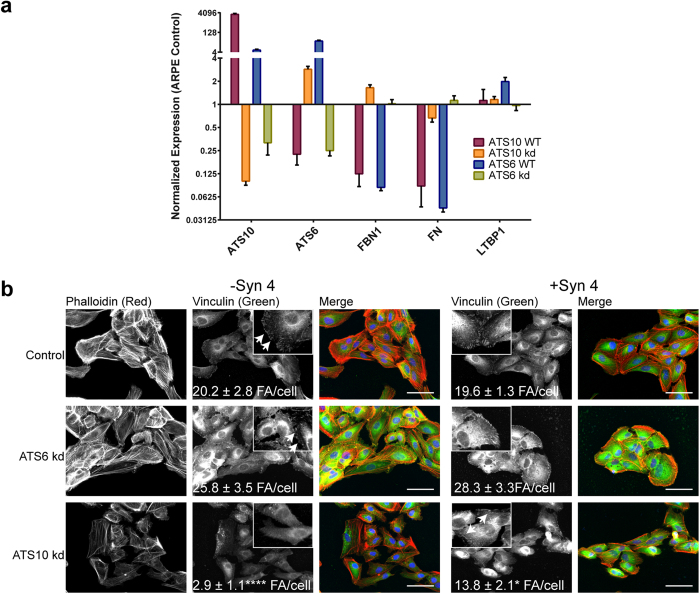
ADAMTS6 and ADAMTS10 can regulate the gene expression of each other. (**a**) RT-qPCR analysis of gene expression for ADAMTS10 and ADAMTS6 overexpression (ATS10 WT and ATS6 WT) and knock-down (kd) in ARPE-19A cells. Relative expression normalized to control cells is shown, and was calculated using Bio-Rad CFX Manager V3.1. Normalized expression is plotted on a Log2 scale (n = 3 for both biological and technical replicates). (**b)** Immunofluorescence microscopy of ARPE-19A cells with siRNA treatment of ADAMTS10 (ATS10 kd) or ADAMTS6 (ATS6 kd). ATS6 kd cells had more focal adhesions than control cells; ATS10 kd cells had significantly fewer focal adhesions than control cells. Transfection with full-length syndecan-4 (+Syn 4) induced focal adhesions in ATS10 kd cells. Statistical significance for deviation from the control values was calculated using 1-way ANOVA with a Bonferroni’s multiple comparisons test using GraphPad Prism V6. Asterisk indicate P values where ****P ≤ 0.0001 (n > 8).

ADAMTS10 or ADAMTS6 was knocked down in ARPE-19A cells, and HDFs, for up to 8 days, by siRNA repeated every 48 hours to ensure maximal knock-down. Knock-downs of ADAMTS10 and ADAMTS6 in ARPE-19A were 90% and 75% respectively. Knockdown of ADAMTS10, resulted in a 2.9 fold increase in gene expression of ADAMTS6. Conversely overexpression of ADAMTS10 resulted in a 4.5 fold decrease in ADAMTS6 gene expression. This suggests a regulatory role of ADAMTS10 for ADAMTS6 expression. Whereas ADAMTS6 expression had a synergistic effect on ADAMTS10 gene expression, overexpression of a ADAMTS6 resulted in a 5.7 fold increase in ADAMTS10 gene expression, while ADAMTS6 knockdown, resulted in a 3.2 fold decrease in ADAMTS10 gene expression (Fig. 2a).

ADAMTS6-depletion cells had prominent adhesions in ARPE-19A cells, and HDFs (Fig. 2b, Supplementary Fig. 2a), whereas ADAMTS10-depleted cells had very few focal adhesions, which may be due to the increased gene expression of ADAMTS6 or that ADAMTS10 is needed to stabilise the focal adhesions. Transfection of ADAMTS10-depleted ARPE-19A cells with syndecan-4 partially rescued focal adhesions (Fig. 2b). The increase in syndecan 4 molecules, may have negated the increased ADAMTS6 resulting in less disruption to focal adhesions in a syndecan-4-dependent manner. The overexpressed syndecan-4 colocalized with increased pericellular fibrillin-1 which may also have increased the stability of focal adhesions (Supplementary Fig. 2b). ADAMTS10 can support focal adhesion formation by negatively regulating ADAMTS6.

#### ADAMTS6 disrupts cell surface HS

**Figure 3:**
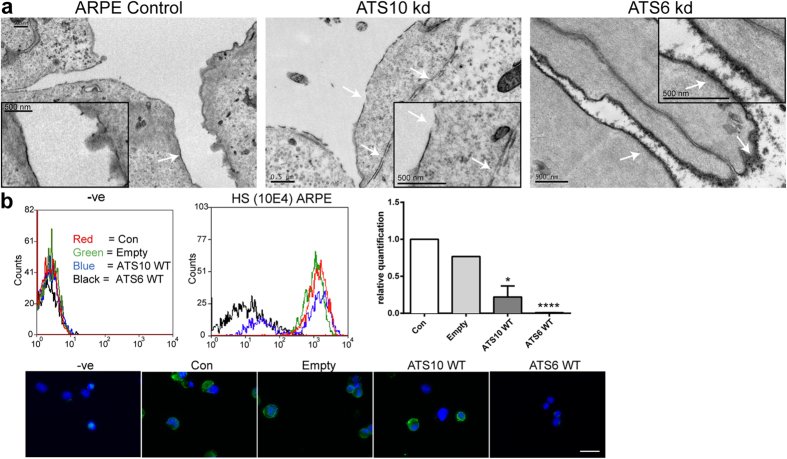
ADAMTS6 and ADAMTS10 can control HS at the cell surface. (**a**) Electron microscopy image of ARPE-19A cells (control and ATS knockdowns) cultured for 2 days; cell surfaces are indicated with white arrows. The scale bars indicate 500 nm. ARPE-19A control and ADAMTS10 siRNA (kd) cells had smooth surfaces, but the ADAMTS6 kd cells had an abundant coating that resembled a thick glycocalyx. (**b**) Flow cytometry showing histograms of cells treated with anti-HS antibody (10E4), and secondary only control (−ve). Flow cytometry showed that ADAMTS6 overexpressing cells (ATS6 WT) cells were depleted of HS. Most ADAMTS10 overexpressing cells (ATS10 WT) retained HS, although some loss was apparent, which has resulted in a histogram with two peaks. These data are also quantitated; asterisks indicate P values where *P ≤ 0.05; ****P ≤ 0.0001. Cells were analysed using BD LSR Fortessa Analyzer. The flow-isolated cells (using Cytospin) are also shown, with HS (mAb 10E4) immunostaining (green) and nuclei were visualized with DAPI (blue). Images were taken with a 40x objective. Scale bar indicates 50 μm.

As it was found that loss of ADAMTS6 and ADAMTS10 may alter the cell surface proteoglycans, investigation of the cell surface using electron microscopy was performed. Electron microscopy (EM) revealed that, whereas ADAMTS10 siRNA-treated cells had no detectable glycocalyx, ADAMTS6 siRNA-treated ARPE-19A cells had a prominent cell surface glycocalyx (Fig. 3a). Conversely, flow cytometry and immunofluorescence microscopy using an anti-HS monoclonal antibody revealed that the ADAMTS6 overexpressing cells had no detectable HS (Fig. 3b). Since HS-containing syndecan-4, with FN (through α5β1 integrin), is required to induce focal adhesions^25^, HS depletion by ADAMTS6 will inhibit adhesions.

#### C-terminal fragments of ADAMTS10 and ADAMTS6 bind focal adhesion molecules and can induce adhesions

**Figure 4:**
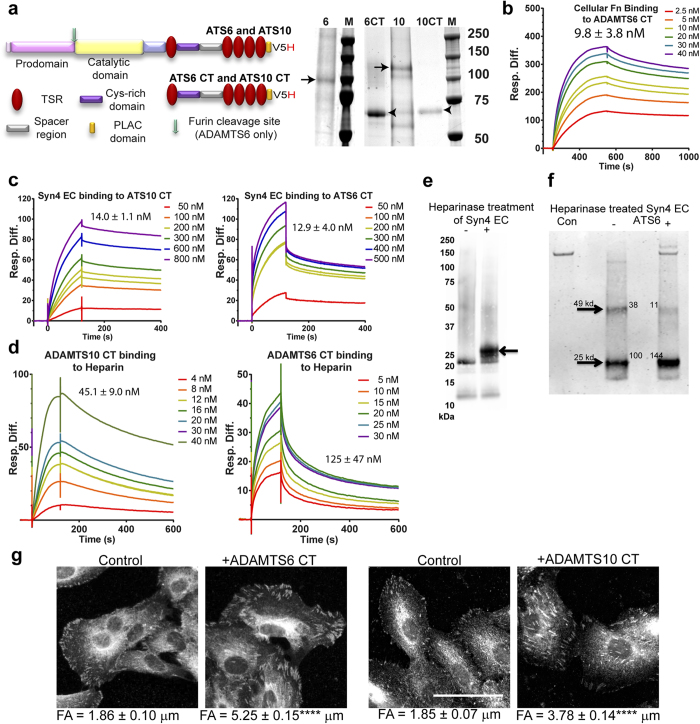
ADAMTS10 and ADAMTS6 interactions with syndecan-4 and modification of focal adhesions. **(a)** Coomassie-stained SDS-PAGE showing purified recombinant full-length ADAMTS6 and ADAMTS10, expressed using the 293-EBNA system^16^. ADAMTS6 was ~100 kDa and ADAMTS10 was ~140 kDa (arrows). The C-terminal regions of ADAMTS6 (6CT) and ADAMTS10 (10CT) were also expressed (arrowheads). Equal amounts of protein were loaded on the gel, however expression levels of the full-length proteins were lower than the C-terminal fragments. Domain structures of full-length molecules and the ADAMTS6 and ADAMTS10 C-terminal region are shown. For purification and detection purposes, all proteins had a C-terminal V5 and His_6_ tag added (V5H). **(b**–**d)** Binding was analysed using Surface Plasmon Resonance, and the response difference (Resp. Diff.) for each experiment was plotted against time (s) **(b)** Biacore sensorgram showing that cellular FN (Sigma #F2518; isolated from human foreskin fibroblasts) binds to the C-terminal region of ADAMTS6. Resp. Diff. is the ADAMTS6-immobilized flow cell minus the control flow cell. The binding constant (KD) indicated and was calculated using 1:1 Langmuir model. (**c**) Biacore sensorgrams showing that ADAMTS10 CT and ADAMTS6 CT bind the ecto-domain of syndecan 4. Resp. Diff. is the syndecan-immobilized flow cell minus the control flow cell. The K_D_ for each interaction is shown. (**d**) Biacore sensorgrams showing that ADAMTS10 CT and ADAMTS6 CT bind heparin. Resp. Diff. is the heparin-immobilized flow cell minus the control flow cell. The K_D_ for each interaction is shown. (**e**) Recombinant syndecan-4 ectodomain was treated with heparinases I, II and III (indicated +) and analysed by SDS-PAGE and western blotting using anti-syndecan-4 antibody. **(f)** Heparinase-treated syndecan-4 ectodomain incubated with full-length recombinant ADAMTS6 (ATS6) overnight at 37 °C. **(g)** Control ARPE-19A cells ± supplementation with purified recombinant C-terminal regions of ADAMTS6, and ADAMTS10 (10 nM) showing focal adhesions (FA) (vinculin stained, B/W). Focal adhesions (FA) lengths were calculated by manually using ImageJ. Adhesions were significantly longer and more prominent in the presence of the C-terminal ADAMTS6 and ADAMTS10 fragment. Statistical significance for deviation from the control values was calculated using 1-way ANOVA with a Bonferroni’s multiple comparisons test using GraphPad Prism V6. Asterisk indicate P values where ****P ≤0.0001. Scale bar indicates 50 μm.

Since active overexpression of ADAMTS6 did not support focal adhesions and overexpression of ADAMTS10 increased focal adhesions, we generated recombinant full-length molecules and C-terminal (starting at TSR1) regions in 293-EBNA cells to investigate HS binding and adhesion-triggering potential. Both C-terminal regions expressed well, but both full-length proteins expressed only at low levels. The apparent molecular weight by SDS-PAGE for ADAMTS6 was ~100 kDa and ADAMTS10 was ~140 kDa (Fig. 4a).

FN induces focal adhesions by binding α5β1 integrin and syndecan-4^26^. Biacore analysis revealed that the monomeric C-terminal region of ADAMTS6 interacted with FN (K_D_ 9.8 ± 3.8 nM), but the corresponding region of ADAMTS10 did not (Fig. 4b; not shown). The C-terminal regions of both ADAMTS10 and ADAMTS6 strongly bound the syndecan-4 ectodomain with K_D_s of 14.0 ± 1.1 nM and 12.9 ± 4.0 nM respectively (Fig. 4c), and HS with K_D_s of 45.1 ± 9.0 and 125 ± 47 nM respectively (Fig. 4d).

Recombinant syndecan-4 extracellular domain (Syn4 EC) was treated with heparinases I, II, III to reduce heterogeneity due to HS. Treatment with heparinase resulted in an increase in a band corresponding to the core protein, and a decrease in higher MW species. This indicated that the recombinant protein is modified with heparin chains (Fig. 4e). The heparinase treated Syn4 EC was incubated with purified recombinant ADAMTS6. Western blot analysis using anti-syndecan-4 antibody showed a 3.5 fold decrease in an SDS-resistant core protein dimer with apparent molecular mass of 49 kDa, and a corresponding increase in the monomeric Syn4 EC core (apparent molecular mass of 25 kDa) when incubated with ADAMTS6 (Fig. 4f). This suggests ADAMTS6 may cleave syndecan 4 close to the C-terminal of the extracellular domain, which contains cleavage sites for MT1-MMP, MMP7 and plasmin^27^.

Supplementation of ARPE-19A cultures with either ADAMTS6 or ADAMTS10 C-terminal regions stimulated adhesions (Fig. 4g) and causing them to be significantly longer. Thus the C-terminal region of both molecules can drive focal adhesion formation at the cell surface, possibly by adhesion to syndecan-4 HS chains, and promote clustering of the proteoglycan. The c- terminal regions of both ADAMTS6 and ADAMTS10 have identical domain structures; both contain heparin binding thrombospondin type 1-like (TSR) repeats, and cysteine rich domains. The cysteine-rich domain of human ADAM12, has been found to support cell adhesion, through to heparin sulphate chains^28^. Clustering of syndecan-4 by the interaction with the heparin binding domain of fibronectin has been previously found to drive focal adhesion formation^29^.

### ADAMTS10 supports but ADAMTS6 inhibits epithelial cell junctions

We and others have shown that fibrillin-1 and FN deposition by epithelial cells is regulated by adherens junctions^20^,^30^. Here, we confirmed the need for tight junctions for microfibril and FN deposition by ARPE-19A cells (Supplementary Fig. 3a), and investigated whether either ADAMTS alters cell junctions in epithelial cultures (ARPE-19A, EpH4).

**Figure 5:**
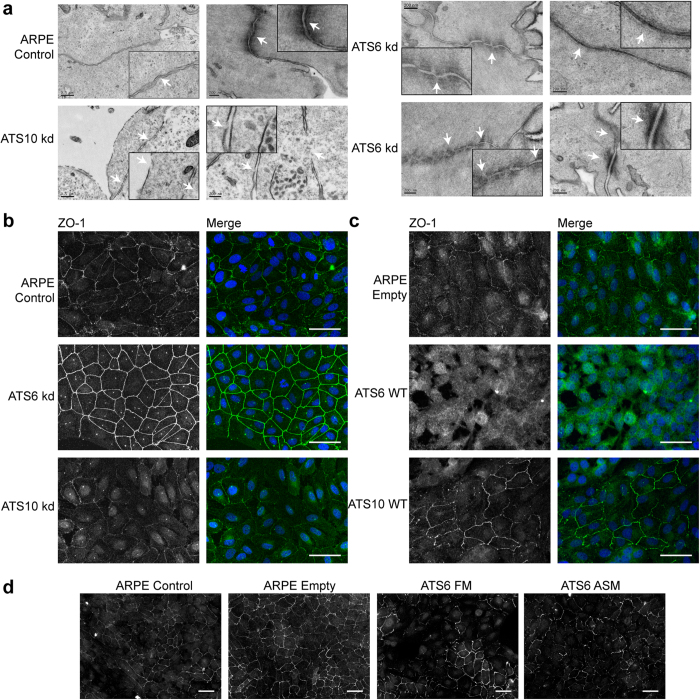
Epithelial cell-cell junctions, and effects of ADAMTS siRNA. (**a**) Electron micrography of ARPE-19A cells cultured for 2 days; cell junctions are indicated with white arrows. The scale bars indicate 0.5 μm (1st column) or 200 nm. Control ARPE-19A cells had few tight junctions but some adherens junctions. ADAMTS10 siRNA cultures had disrupted membranes and no tight junctions. ADAMTS6 siRNA cultures had numerous tight, adherens and desmosome junctions. **(b**–**d)** Immunofluorescence microscopy of ARPE-19A cultured on glass coverslips for 4 days. **(b)** Cells were treated with siRNAs to down-regulate ADAMTS6 (ATS6 kd) or ADAMTS10 (ATS10 kd). **(c)** Cells with lentivector overexpression of ADAMTS10 (ATS10 WT) or ADAMTS6 (ATS6 WT). After fixation, cells were stained for ZO-1 (B/W, green), and nuclei were visualized with DAPI (blue). Images were taken with a 40x objective. Scale bar indicates 50 μm. **(d)** Cells with lentivector overexpression of ADAMTS6 mutants (ATS6 FM and ATS6 ASM). After fixation, cells were stained for ZO-1. Images were taken with a 20x objective. Scale bar indicates 50 μm.

EM revealed that ARPE-19A control cells, after 2 days in culture, had adherens and tight junctions (Fig. 5a). After ADAMTS6 siRNA, cell-cell junctions, including tight, adherens and desmosomes, were even more abundant than controls. Upon ADAMTS10 siRNA, cell-cell interfaces had disconnected membranes and no detected tight junctions.

Immunofluorescence microscopy of ARPE-19A and EpH4 cultures revealed that ADAMTS10 knockdown inhibited tight junctions, as assessed by disrupted ZO-1 (Fig. 5b, Supplementary Fig. 3b), correlating with EM images of disconnected cell membranes (see Fig. 5a). As expected, ADAMTS6 knockdown led to intense ZO-1 immunostaining at cell-cell junctions (Fig. 5b).

Overexpression experiments showed the converse. Enhanced ADAMTS10 expression increased tight junctions, but cells overexpressing ADAMTS6 showed no tight junctions in ARPE-19A cells (Fig. 5c) and reduced tight junction in EpH4 cells (Supplementary Fig. 3c). ARPE-19A cells overexpressing ADAMTS6 furin cleavage site mutant (ATS6 FM), and ADAMTS6 active site mutant (ATS6 ASM) did not have a reduction of tight junctions (Fig. 5d).

Thus, ADAMTS10 supports but ADAMTS6 disrupts cell-cell junctions, and this disruption is due to the enzymic activity of ADAMTS6. These experiments together show that ADAMTS10 is required for, but ADAMTS6 disrupts epithelial cell-cell junctions and HS-rich focal adhesions in epithelial and mesenchymal cells.

### ADAMTS6 is processed and catalytically active

**Figure 6:**
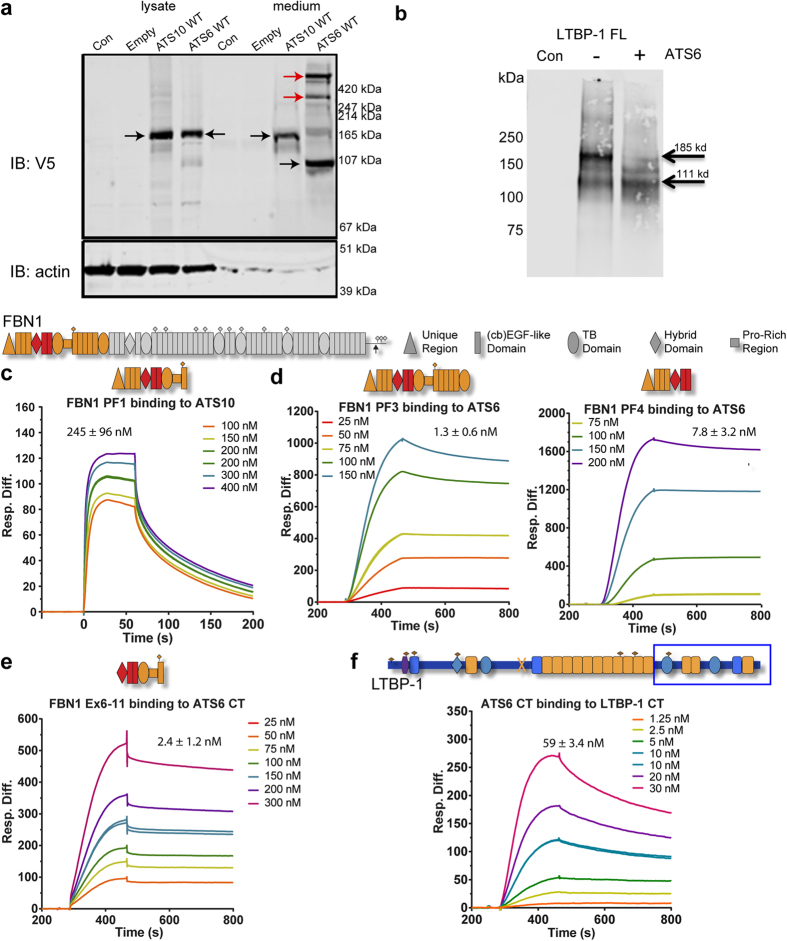
ADAMTS6 but not ADAMTS10 is processed and catalytically active, binds microfibrillar proteins. (**a**) Western blot against V5 tag of ADAMTS10 (ATS10 WT) and ADAMTS6 (ATS6 WT) overexpressed proteins using lentivirus. The SDS-PAGE was run under reducing conditions. Both full-length molecules expressed well in this system. Unprocessed ADAMTS10 was present in medium and cell lysates, whereas ADAMTS6 was processed in medium (black arrows). Some larger bands may be aggregated forms of the processed molecule (red arrow), which are possibly trimeric and tetrameric in nature. (**b**) Full-length LTBP-1 was treated with full-length recombinant ADAMTS6 overnight at 37 °C (molar ratio LTBP-1:ADAMTS6 3:1). Western blot analysis using anti-LTBP-1 C-terminal antibody showed a reduction in full-length LTBP-1 with a relative molecular mass of 185 kDa, and an increase in intensity of a 111 kDa degradation product. The cleavage site of BMP-1^44^, indicated by ‘X’ on LTBP-1 domain map (Fig. 6F), also generates a 110 kDa C-terminal fragment. Control lane (con) contains ADAMTS6 only. **(c**–**f)** Biacore sensorgrams showing that: **(c)** Full-length ADAMTS10 binds the fibrillin-1 PF1 fragment, and **(d)** full-length ADAMTS6 binds to recombinant N-terminal fibrillin-1 (PF3 and PF4). (**e**) To further resolve the binding regions, it was found that ADAMTS6 CT regions (Fig. 4) bound fibrillin-1 Ex6–11 fragment^16^. The binding of C-terminal ADAMTS6 was mapped to exons 6–8 of FBN1, indicated in red. **(f)** C-terminal ADAMTS6 binds to LTBP-1 C-terminal region (indicated by box). Binding was analysed using Surface Plasmon Resonance, and the response difference (Resp. Diff.) for each experiment was plotted against time (s). Resp. Diff. is the ligand-immobilized flow cell minus the control flow cell.

To investigate further the differential effects of ADAMTS10 and ADAMTS6 on microfibrils, we examined further whether they are processed and catalytically active. Using lentiviral expression in ARPE-19A cells, full-length monomeric ADAMTS6 and ADAMTS10 were secreted, yet only ADAMTS6 was processed extracellularly (Fig. 6a). Secreted ADAMTS6 was also found to form SDS resistant multimers, which are possibly trimeric and tetrameric in nature. It has been recently reported that ADAMTS5 also forms oligomers via the catalytic and disintegrin-like domains, with a nominal molecular weight of ~400 kDa^31^. Oligomerisation of ADAMTS5 was required for aggrecanase activity, which maybe also the case for the enzymic activity of ADAMTS6. Following on from the cleavage of syndecan 4 and data from the ADAMTS6 mutants which confirmed that ADAMTS6 was catalytically active, purified recombinant ADAMTS6 was incubated with full-length LTBP-1 (Fig. 6b). LTBP-1 which colocalises with microfibrils^32^ was also found to be partially cleaved by ADAMTS6.

### ADAMTS6 binds microfibril molecules more strongly than ADAMTS10

ADAMTS10 was reported to bind two regions of fibrillin-1, an N-terminal site and a second site towards the C-terminus^3^. Our N-terminal fibrillin-1 fragment, PF1 (encoded by exons 1–11^16^) interacted with ADAMTS10 with relatively low affinity (K_D_ 245 ± 96 nM) (Fig. 6c). We did not detect significant binding to any other of our overlapping fragments which encompass full-length fibrillin-1^16^.

We identified a high affinity interaction between full-length ADAMTS6 and N-terminal fibrillin-1 PF3 and PF4 fragments (K_D_s 1.3 ± 0.6 nM and 7.8 ± 3.2 nM, respectively) (Fig. 6d). The binding region of ADAMTS6 on fibrillin-1 was mapped using a deletion series of N-terminal fragments^16^; the smallest fragment that strongly bound the C-terminal region of ADAMTS6 (K_D_ 2.4 ± 1.2 nM) comprised domains encoded by exons 6–11 (Ex6–11) (Fig. 6e). As ADAMTS6 bound fragments PF4 (encoded by exons 1–8) and Ex6–11, the binding site is thus mapped to the domains encoded by exons 6–8 (Fig. 6d,e). ADAMTS6 also bound a modified fibrillin-1 PF3 fragment (encoded by exons 1–15) with a WMS mutation (exon 9–11 deletion^33^) (not shown).

We also explored whether ADAMTS10 or ADAMTS6 bind microfibril-associated LTBP-1 which regulates latent TGFβ. A C-terminal region of LTBP-1 which binds N-terminal fibrillin-1^32^ bound the C-terminal fragment of ADAMTS6 (K_D_ 59 ± 3.4 nM) (Fig. 6f), but C-terminal ADAMTS10 did not bind significantly to LTBP-1 (not shown).

Thus ADAMTS6 binds more strongly to microfibrillar molecules than ADAMTS10.

### ADAMTS10 supports but ADAMTS6 inhibits microfibril deposition

Having shown that ADAMTS6 differs from ADAMTS10 by strongly binding and cleaving microfibrillar molecules, and by disrupting focal adhesions and cell-cell junctions, we compared how both molecules influence microfibril deposition.

**Figure 7:**
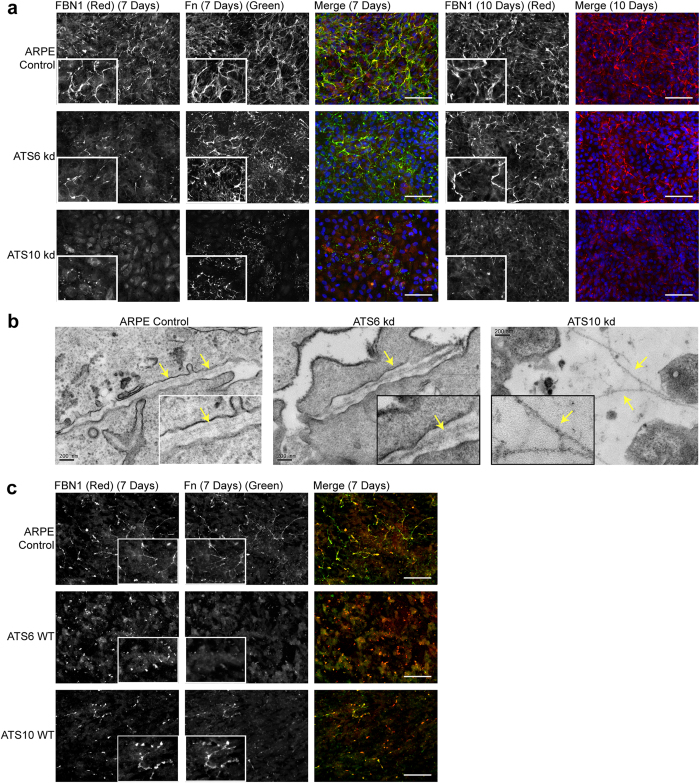
Effects of ADAMTS knockdown on fibrillin and fibronectin deposition. (**a**) Immunofluorescence microscopy of ARPE-19A cells cultured on glass coverslips for 7 and 10 days. Cells were treated with siRNAs to down-regulate either ADAMTS6 (ATS6 kd) or ADAMTS10 (ATS10 kd). After fixation, cells were stained for fibrillin-1 (FBN1; B/W, red) and FN (B/W, green), and nuclei were visualized with DAPI (blue). Shown are images taken with a 40x objective, plus a 2x zoomed area of the fibrillin-1 and FN stained images (inset). Scale bars indicate 50 μm. **(b)** Electron micrography of ARPE-19A cells cultured for 2 days; fibrillin microfibrils are indicated with yellow arrows. The scale bars indicate 200 nm. Control ARPE-19A and ADAMTS6 siRNA cultures contained microfibril arrays, but in ADAMTS10 siRNA cultures only single microfibrils were seen. **(c)** Immunofluorescence microscopy of ARPE-19A cells cultured on glass coverslips for 7 days. Cells with lentivector overexpression of ADAMTS10 (ATS10 WT) or ADAMTS6 (ATS6 WT) were stained for fibrillin-1 (B/W, red) and FN (B/W, green). Shown are images taken with a 40x objective, plus a 4x zoomed area of the FBN1 and FN stained images (inset). Scale bars indicate 50 μm.

Knockdown studies revealed that ARPE-19A cultures required ADAMTS10 for microfibril deposition. Immunofluorescence microscopy after 7 and 10 days culture (anti-fibrillin-1 antibody HPA021057; anti-FN antibody F6140) showed that ADAMTS10-depleted cultures had no detectable microfibril arrays compared to control and ADAMTS6-depleted cells which deposited microfibrils, although there was a slight reduction in microfibril density (Fig. 7a). By EM after 2 days, ADAMTS10 siRNA cells had assembled a few individual microfibrils but no microfibril arrays were detected, unlike control and ADAMTS6 siRNA cells which deposited microfibril arrays (Fig. 7b). Overexpression studies showed that enhanced ADAMTS6 expression resulted in a loss of microfibrils deposition, and no fibronectin deposition. However enhanced ADAMTS10 expression did result in some microfibril and fibronectin deposition although it was at a reduced level compared with the control (Fig. 7c). Overexpression of ADAMTS6 and ADAMTS10 resulted in a loss of fibrillin-1 gene expression (12 fold and 8 fold respectively) and a loss of fibronectin gene expression (22 fold and 12 fold respectively) (Fig. 2a).

Our data imply that ADAMTS6 disrupts microfibril deposition in epithelial cells by depleting HS and disrupting focal adhesions and cell junctions. In contrast ADAMTS10 supports cell-cell and HS-rich cell-matrix interfaces involved in microfibril deposition, possibly through control of the of ADAMTS6 gene expression.

## Discussion

We have shed light on the functional relationship between fibrillin-1 and ADAMTS10 indicated by their genetic linkage to WMS, by comparing ADAMTS10 with its homologue ADAMTS6. This is the first time a detailed study on the ADAMTS6 protein has been carried out, and it was found to have a major role in focal adhesion and tight junction formation and can alter the deposition of FBN1 microfibrils in epithelial cells. One possible role for ADAMTS10 could be as a regulator of ADAMTS6 gene expression. It was found that knockdown of ADAMTS10 gene expression resulted in an increase in ADAMTS6 gene expression, while the opposite was observed when ADAMTS10 was overexpressed. Overexpression of ADAMTS6 resulted in a corresponding increase of ADAMTS10 gene expression, and knockdown of ADAMTS6 resulted in lower ADAMTS10 gene expression. If increased gene expression of ADAMTS6 up-regulates ADAMTS-10 and increased gene expression of ADAMTS10 downregulates ADAMTS6, this suggests a negative feedback interaction of ADAMTS10 on ADAMTS6. The gene expression of ADAMTS10 is very low in ARPE-19 cells, but despite this, knockdown of ADAMTS10 had prominent effects on focal adhesions, tight junctions and microfibril deposition. Thus, its role as a gene regulator of ADAMTS6 may be more important than direct interactions with these processes.

**Figure 8:**
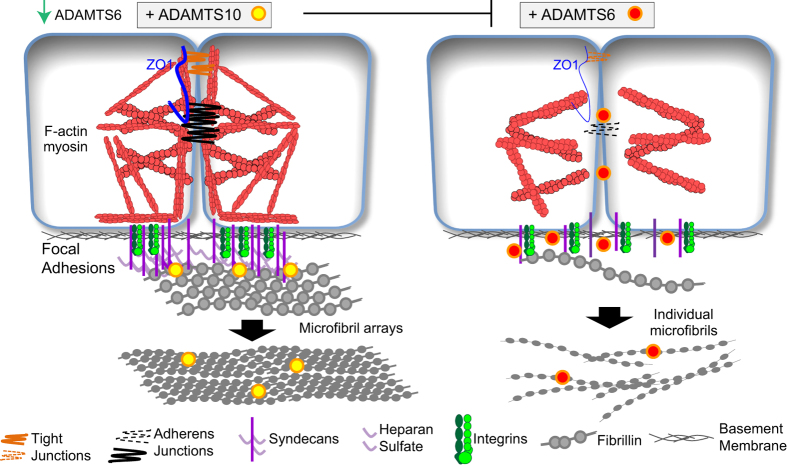
Model of how ADAMTS6 and ADAMTS10 can regulate cell and focal adhesions and alter deposition of fibrillin microfibril arrays by retinal epithelial cells. In the presence of ADAMTS10, which down regulates ADAMTS6 gene expression, cells can form adherens and tight junctions, and focal adhesions, which together support the formation of ordered microfibril arrays by enhancing fibrillin-1 multimerization and the organisation of microfibrils within bundles. When ADAMTS10 is depleted, and ADAMTS6 is consequently enhanced, cell-cell junctions are lost and cell surface HS is reduced. Consequently, fibrillin-1 multimerization and microfibril formation are reduced, and bundles are disorganized. The cytoskeleton also becomes poorly arranged.

Our data show that increased expression of ADAMTS6 results in loss of tight junctions, focal adhesions and fibrillin-1 and fibronectin deposition. ADAMTS10 supports HS-rich cellular interfaces involved in the deposition of microfibril arrays; this could be by direct interaction of ADAMTS10 with microfibrils or control of ADAMTS6 gene expression. We have previously shown that loss of syndecan-4 from ARPE-19A cells resulted in a loss of microfibril deposition^20^; therefore increased expression of ADAMTS6 which reduces focal adhesions may be detrimental to microfibril deposition (Fig. 8).

Despite low expression by retinal epithelial cells, ADAMTS10 but not ADAMTS6 supported the HS-rich cell interfaces needed for microfibril deposition. Since syndecan-4 partially rescued ADAMTS10 depletion, syndecan-4 is implicated in this function. We confirmed that ADAMTS6 was processed and catalytically active, and that it depleted cell surface HS, and blocked focal adhesions. Cleavage of syndecan-4 by ADAMTS6 suggests this could a possible mechanism of action, for the loss of focal adhesions. This process was reversed when the overexpressed ADAMTS6 was made catalytically in-active, either by disrupting the furin cleavage site or mutating the metalloprotease active site motif. Conversely, loss of ADAMTS6 enhanced cell surface HS and focal adhesions. ADAMTS6 may thus be added to the ADAMTS members that are receptor sheddases^34^,^35^.

HS interactions with fibrillin-1 are enhanced by multimerization (fibrillin-1 exists *in vivo* as bundles of microfibrils), whilst fibrillin-1 (TB5 domain) HS interactions are inhibited by WMS, AD and GD mutations^10^. Mutations in the adjacent fibrillin-1 TB4 RGD containing region (which is needed for focal adhesions^19^) perturb microfibril arrays^36^, leading to stiff skin syndrome. We suggest that the fibrillin-1 focal adhesion-inducing region, integrin- (TB4) and HS-binding (TB5) domains, controls deposition of bundles of microfibrils with ADAMTS10 supporting HS-dependent interactions.

ADAMTS10 can bind fibrillin-1 and colocalise with microfibrils^3^. However, we found that, unlike ADAMTS6, ADAMTS10 bound fibrillin-1 only relatively weakly and not significantly to microfibril-associated LTBP-1, so it is unlikely to block ADAMTS6 binding to microfibrils and thereby prevent degradation. However, ADAMTS6 has the potential to form a complex with LTBP-1 and fibrillin-1, which may affect microfibril integrity. We did not detect the C-terminal fibrillin-1 binding site for ADAMTS10 reported by Kutz *et al*.^3^, which may reflect fragment differences.

We showed that epithelial adherens junctions support microfibril deposition, and that epithelial-mesenchymal status alters dependence on FN for microfibril deposition^20^. Here, ADAMTS10 differed strikingly from ADAMTS6 in its effects on cell-cell junctions, with ADAMTS6 disrupting but ADAMTS10 enhancing tight junctions. Since junctions were enhanced when ADAMTS6 was depleted or mutated, ADAMTS6 may degrade them. Indeed, other ADAMs can cleave cadherins^37^. Disruption of tight junctions was not seen with overexpression of the two ADAMTS6 mutants, confirming that the metalloprotease activity is responsible for tight junction disruption. In summary, ADAMTS6 affects microfibril deposition through HS-rich cellular interfaces, and control of gene expression which may be modulated by ADAMTS10.

## Methods

### Cell culture

ARPE-19A cells were purchased from the American Tissue Culture Collection (CRL-2302). Cells designated ARPE-19A were batch 58280268, and confirmed to be ARPE-19A cells by short tandem repeat analysis^20^. EpH4, murine mammary epithelial cells, were provided by Dr. A. Gilmore (Manchester). Human dermal fibroblasts (HDFs) were purchased from ThermoFisher Scientific and were from a 31 year old female. HDFs were maintained in Dulbecco’s Modified Eagle’s Medium (DMEM; Sigma-Aldrich), ARPE-19A in DMEM-F12 1:1 (Lonza) and both supplemented with 10% (v/v) fetal calf serum (FCS; ThermoFisher Scientific), 1% L-glutamine, 100 U/ml penicillin/streptomycin at 37 °C in 5% CO_2_, and were passaged at confluency.

### Antibodies

The primary antibodies used for immunofluorescence microscopy, western blotting and/or flow cytometry, were: FN (FN-3E2 and F6140, Sigma-Aldrich; 1:200), fibrillin-1 (HPA021057, Sigma-Aldrich, 1:200; mAb and 11C1.3, Abcam, 1:200), HS (10E4, Seigagaku, Japan; 1:200), N-cadherin (Cell Signaling mAb #13116, 1:200), syndecan-4 (SantaCruz 5G9, sc-12766, 1:40), FAK (Santa Cruz c-20, sc-558, 1:1000), pY397FAK (Abcam ab4803, 1:1000), ZO-1 (T11, Millipore; 1:200), vinculin (hVin-1, Sigma-Aldrich; 1:400), anti-V5 (MCA1360GA; Bio-Rad; 1:2500), anti-β-actin (mAbAC-74, Sigma-Aldrich) as loading control for western blots. Phalloidin conjugated with Alexa 594 (ThermoFisher Scientific; 1:1000) was used to stain the actin cytoskeleton. Secondary antibodies used immunofluorescence microscopy and flow cytometry, were purchased from ThermoFisher Scientific: Donkey anti-mouse Alexa Fluor 350 (A10035), 488 IgG (A21202), 488 IgM (A21042); Donkey anti-rabbit Alexa Fluor 488 (A21206), 594 (A21207); Donkey anti-rat Alexa Fluor 488 (A21208), 594 (A21209). All cell lines expressing GFP were stained using the blue (350 nm or dapi) and red (594 nm) channels, then false coloured green to be consistent with non-GFP expressing cell lines.

### siRNA transfections

Cells were transfected using lipofectamine RNAiMAX reagent (ThermoFisher Scientific), according to the manufacturer’s protocol, as described^20^. Briefly, cells were plated in 6-well plates or 24-well plates with glass coverslips (ARPE-19A, 300,000 per 6-well; 60,000 per 24-well; HDFs, 250,000/50,000; EpH4 50,000/10,000) and left to adhere for 2 hours. 40 pmol RNAi duplex was added to 500 μl Optim-MEM medium (ThermoFisher Scientific) prior to addition of 2.5 μl Lipofectamine RNAiMAX per well of a 6-well plate or 4 × 2-wells, with Lipofectamine RNAiMAX-only controls, and incubation for 15 mins. Transfection mix was added to the cells, giving a final RNAi duplex concentration of 10 nM. Cells were cultured for up to 10 days, with repeated RNAi duplex transfection taking place at 2-day intervals. RNA and protein lysates were harvested at 4 days. RNAi duplexes used were: Qiagen, ADAMTS6 Hs_ADAMTS6_7, ADAMTS10 Hs_ADAMTS10_8; ThermoFisher Scientific, ADAMTS6: siRNA (2) ID-113433; siRNA (3) ID-114002; siRNA (4) ID-104129. DNA and RNA oligonucleotides were purchased from MWG Operon.

### Lentiviral overexpression

Stably expressing epithelial cells (ARPE-19A, EpH4) were generated using an established lentiviral method (SBI Systems Bioscience). cDNA sequences used for lentiviral overexpression were based on ADAMTS10 variant 1 (NCBI Ref. Seq. NM_030957.3) and ADAMTS6 variant 1 (NCBI Ref. Seq. NP_922932.2). ADAMTS constructs were created that contained an N-terminal BM40 signal peptide and a C-terminal V5 tag followed by a hexahistidine tag, and were subcloned into the viral expression vector pCDH-EF1-T2A-copGFP vector (provided by Dr. A. Gilmore). ADAMTS6 full-length (ATS6 WT) is encoded by residues 22-1117, and ADAMTS10 full-length (ATS10 WT) is encoded by residues 26-1103. Two further mutants of ADAMTS6 were created; the first had a site-directed mutation in the metalloprotease active site motif (ATS6 ASM), the second had a site-directed mutation in the furin cleavage site (ATS FM). The ATS6 ASM site-directed mutation was E404A, which changed the active site motif from H**E**IGHNFGMNHD to H**A**IGHNFGMNHD. The ATS6 FM site-directed mutation was R241A, which changed the furin cleavage site from **R**QKR to **A**QKR. Mutations were created using Q5^®^ Site-Directed Mutagenesis Kit (NEB), and fully sequenced before use. HEK293T cells were co-transfected with psPax2, pMD2.G packaging as well as a target pCDH-EF1-T2A-copGFP vector. Production of virus particles was induced by addition of 10 mM sodium butyrate (Millipore). 24 h post-transfection, virus particles were sterile-filtered and concentrated by centrifugation (21,000 rcf for 4 h), and virus pellets stored at −80 °C. Epithelial cells were infected with virus using 50 μg/ml Protamine sulphate (Sigma). Cells were passaged followed by fluorescence-activated cell sorting (FACSAria Fusion, Beckon-Dickenson). For all experiments, only lowest 20% expressing cells were selected.

### Immunofluorescence microscopy

Cell organization and ECM deposition were analysed by indirect immunofluorescence microscopy, as described previously^20^. Some cells were stained with phalloidin (as above). Images were collected at room temperature on an Olympus BX51 upright microscope using 20x or 60x objectives and captured using a Coolsnap ES camera (Photometrics) through MetaVue Software (Molecular Devices). Specific band pass filter sets for DAPI, FITC, Texas Red, Cy3 and Cy5 were used to prevent bleed through from one channel to the next. Images were processed and analysed using ImageJ (http://rsb.info.nih.gov/ij). Spheroids were harvested from 96 well plates and placed into 1.5 ml low binding Eppendorf tubes (Eppendorf). Media was aspirated and spheroids were fixed in 4% PFA and 1% Triton X100, and dehydrated and permeabilized using increasing, then decreasing concentrations of methanol as described^38^,^39^. Primary and secondary antibodies were incubated overnight and DAPI was added with secondary antibodies. Spheroids were washed in PBS/0.1% Triton X-100, before imaging by suspending spheroids in a well of a 6-well plate in 2ml PBS. Images were collected on a Leica TCS SP5 AOBS upright confocal using a 20x/0.50 HCX Apo L dipping objective.

### Peptide inhibition of cadherin junctions

ARPE-19A cells, with or without ADAMTS6 knock-down, were cultured for 2 days before the overnight addition of anti-cadherin peptide (1 mM) or TNFα (5 ng/ml), as described (Baldwin *et al*.)^20^; the peptide was supplied by Dr. C. Ward, Manchester). Epithelial cell-cell junctions, cytoskeleton and microfibril deposition were compared by indirect immunofluorescence microscopy (as above).

### Western blotting

Western blotting was carried out as described^20^. Blots were probed with anti-FN (mouse mAb FN-3E2, Sigma-Aldrich), anti-fibrillin-1 (HPA021057, Sigma-Aldrich) anti-FAK (rabbit Santa Cruz c-20, sc-558), or anti-pY397FAK (rabbit Abcam ab4803), overnight at 4 °C. Blots were developed with horseradish peroxidase secondary antibodies or IR dye conjugated antibodies (Li-COR Biosciences), and analysed using a Chemidoc MP (Bio-Rad) or Odyssey Clx scanner. Band intensities were quantified using Image Lab (Bio-Rad) or Image Studio software (Li-COR Biosciences). The overexpressed protein was concentrated by addition of nickel chelated sepharose, and then detected by western blotting using anti-V5 tag antibody.

### Real-time quantitative PCR (qPCR)

RNA was isolated from cells using ReliaPrep™ RNA Cell Miniprep System (Promega). Up to 2 μg RNA was used to generate cDNA, using a cDNA synthesis kit (Bioline). RT-qPCR analysis was carried out using CFX96/384 instruments (Bio-Rad) and the GoTaq qPCR Mastermix Kit (Promega). RT-qPCR primer sequences are listed in Supplementary Table 1 and were purchased from Eurofins. Due to very low expression levels, ADAMTS10 gene expression analysis was performed using TaqMan probes (Hs01548644_g1 (ADAMTS10), ThermoFisher Scientific), with matching reference gene primers. Expression analysis used CFX Manager Software v3.1 (Bio-Rad), with samples normalized to a combination of TATA box binding protein (TBP) and glyceraldehyde 3-phosphate dehydrogenase (GAPDH) expression. Gene expression data are in Supplemental Information.

### Electron microscopy

ARPE-19A cells were grown on Aclar film for 3 days (with RNAi duplex transfection at day 0) before fixation with 2.5% glutaraldehyde and 4% paraformaldehyde in 0.1 M cacodylate buffer, postfixed with 1% osmium tetroxide for 1 hour, and treated with 1% tannic acid for 1 hour and with 1% uranyl acetate for 1 hour. Samples were dehydrated with alcohol series and embedded in TAAB LV resin. Ultrathin en face sections were cut at a Reichert Ultracut S Ultramicrotome, at basal and upper cell layers, and contrasted with lead citrate. Sections were viewed with an FEI Tecnai Biotwin 12 microscope at 100 kV accelerating velocity.

### Flow cytometry

Flow cytometry samples were prepared as follows: ARPE-19A cells untreated, or stably expressing empty vector, ADAMTS10 or ADAMTS6 were cultured for 24 hours and then detached using cell dissociation buffer (Gibco). Cells (5 × 10^5^) were trypsinized, resuspended in serum free medium and left at room temperature for 30 min in order to allow recovery of cell surface proteins. Following blocking with bovine serum albumin (BSA; Sigma-Aldrich), primary antibodies (anti-HS-10E4) were added to cells, followed by 20 min incubation at 4 °C. Cells were washed twice with PBS and subsequently, Alexafluor 594 or 647 secondary antibodies were added, followed by 20 min incubation at 4 °C and two PBS washes. Subsequently, cells were analyzed using BD LSR Fortessa Analyzer. After flow cytometry analysis, cells were fixed with 1% PFA and cytospan on coverslips (800 g for 5 min) followed by immunofluorescence microscopy.

### Recombinant protein expression

Recombinant human fibrillin-1 fragments were expressed using the mammalian expression vector pCEP-pu/AC7, modified with an N-terminal His6 tag, in 293-EBNA cells^16^,^19^,^40^,^41^. cDNA sequences used for recombinant protein expression were based on ADAMTS10 variant 1 (NCBI Ref. Seq. NM_030957.3) and ADAMTS6 variant 1 (NCBI Ref. Seq. NP_922932.2). Recombinant human ADAMTS6 and ADAMTS10 were expressed using the mammalian expression vector pCEP-pu/AC7 modified with a C-terminal V5-His6 tag, in 293-EBNA cells. ADAMTS6 and ADAMTS10 were cloned from RNA extracted from ARPE-19A cells; ADAMTS6 full-length is encoded by residues 22-1117, and ADAMTS6 C-terminal region starts at the 1st TSR domain and is encoded by residues 558-1117. ADAMTS10 full-length is encoded by residues 26-1103, and ADAMTS10 C-terminal region starts at the 1st TSR domain and is encoded by residues 547-1103 (Fig. 4a). Syndecan-4 ecto-domain was expressed using the mammalian expression vector pCEP-pu/AC7 modified with a C-terminal His_10_ tag, in 293-EBNA cells. Syndecan-4 ectodomain was cloned from RNA extracted from ARPE-19A cells and is encoded by residues 19-143 (ESI….IFE). LTBP-1 was expressed using the mammalian expression vector pCEP-pu/AC7 modified with a C-terminal His_6_ tag and 293-EBNA cells, as described^32^,^42^. All fragments were fully sequenced prior to transfection. All proteins were purified using immobilized metal ion affinity chromatography (IMAC) (HisTrap FF GE Healthcare), under high salt conditions (0.5M NaCl). Fibrillin-1, syndecan-4 and LTBP-1 fragments were further purified using size-exclusion chromatography (Superdex 200 or Superose 6, GE Healthcare). Purity of recombinant proteins was analysed using SDS-PAGE with coomassie blue, and protein concentrations were calculated using BCA Protein Assay (Pierce).

### Biacore interaction analysis

For heparin kinetic binding studies, a heparin oligosaccharide comprising 20 sugar moieties (dp20) (Iduron, UK) was biotinylated via oxidized cis-diol groups and immobilized onto SA sensor chips (GE Healthcare), as described^41^. The heparin oligosaccharide was used at 1 μM, and 500 Response Units (RU) were immobilized. All binding experiments were performed in 10 mM HEPES pH 7.4, 0.1 M NaCl, 0.5 mM CaCl_2_ and 0.005% surfactant P20 (designated HBS-P). All recombinant proteins used for surface plasmon resonance experiments were purified by IMAC, as described above. Full-length ADAMTS6, ADAMTS6 C-terminal region, full-length ADAMTS10, ADAMTS10 C-terminal region, and LTBP-1 C-terminal region were immobilized by amine coupling onto CM5 sensor chips (GE Healthcare) in 50 mM sodium acetate buffer pH 5.5 at 30 μg/ml, giving typical immobilization of 3000 response units (RU) as described^43^. For kinetic studies, protein fragments were injected at a flow rate of 30 μl/min and regeneration was performed by dual injections of 0.8 M NaCl. Binding was either calculated using 1:1 binding kinetics (Biacore T200 Evaluation Software V2.0) or independently using equilibrium analysis. Equilibrium response was plotted against concentration, and non-linear regression was used to calculate K_D_ using the equation for one site binding.

## Additional Information

**How to cite this article**: Cain, S. A. *et al*. ADAMTS-10 and -6 differentially regulate cell-cell junctions and focal adhesions. *Sci. Rep.* **6**, 35956; doi: 10.1038/srep35956 (2016).

## Supplementary Material

Supplementary Information
