# Supplementary material for: ADAMTS-10 and -6 differentially regulate cell-cell junctions and focal adhesions
Source: Sci Rep. 2016 Oct 25;6:35956. doi: 10.1038/srep35956 (PMC5078793; doi:10.1038/srep35956)
Supplement: Supplementary Information [file srep35956-s1.pdf]

ADAMTS-10 and -6 differentially regulate cell-cell junctions and focal adhesions

Stuart A. Cain≠, Ewa J. Mularczyk≠, Mukti Singh, Teresa Massam-Wu, Cay M. Kielty*

Wellcome Trust Centre for Cell-Matrix Research, Faculty of Biology, Medicine and Health, University of Manchester, Manchester M13 9PT, United Kingdom.

Supplementary Material

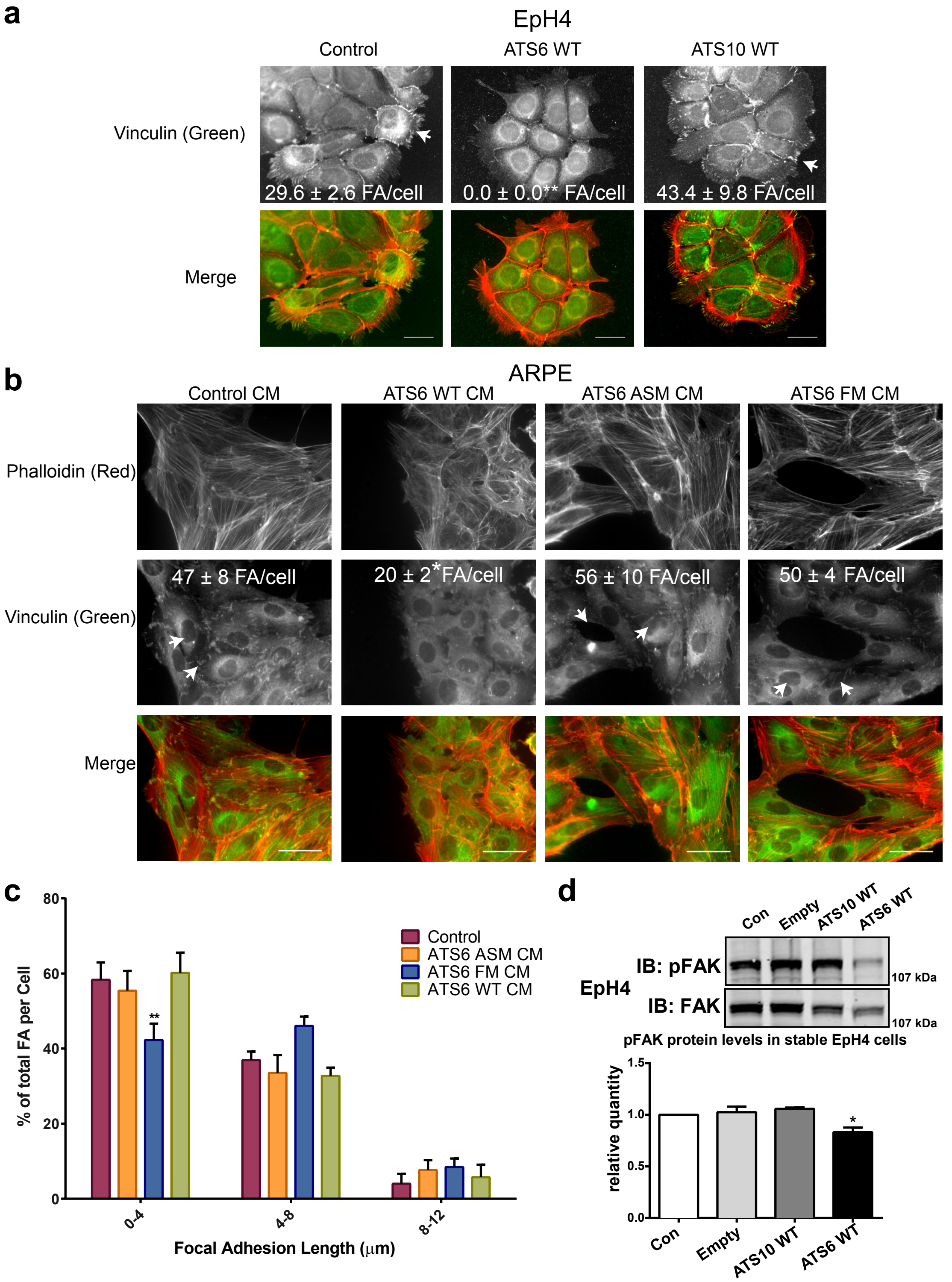

Supplementary Figure 1. Effects of ADAMTS6 and 10 overexpression and knock-down on focal adhesions.
(a) Immunofluorescence microscopy of EpH4 cells overexpressing ADAMTS6 and ADAMTS10. Focal adhesions were visualised by immune staining of vinculin (B/W, green) and phalloidin (B/W, red) on glass coverslips after 3 days. Shown are cells overexpressing ADAMTS6 wild type (ATS6 WT) and ADAMTS10 wild type (ATS10 WT). Control cells were infected with lentivirus containing an empty vector control. EpH4 cells overexpressing ADAMTS6 wild-type protein had no detectable focal adhesions; however more focal adhesions per cell were detected with ADAMTS10 wild-type (ATS10 WT). Examples of focal adhesions are indicated by arrows. The number of focal adhesions (FA) per cell was calculated by manual counting using ImageJ (indicated) (n>8). Images were taken with a 40x objective. Scale bars indicate 50 μm. **(b)** Immunofluorescence microscopy of ARPE-19 cells after the addition of conditioned media (CM) from ARPE-19 cells over-expressing ADAMTS6 and ADAMTS10 (Fig 1A). Focal adhesions were visualised by immune staining of vinculin (B/W, green) and phalloidin (B/W, red) on glass coverslips 1 day after addition of CM (day 3). Shown are cells with after the addition of conditioned media from cells overexpressing ADAMTS6 (ATS6 WT, ATS6 ASM (active site mutant), ATS6 FM (furin cleavage site mutant)) and ADAMTS10 wild type (ATS10 WT). Control cells had addition of CM from empty vector control cells. Conditioned media from ATS6 WT cells has significantly fewer focal adhesions (n>5). Examples of focal adhesions are indicated by arrows. Scale bars indicate 50 μm. **(c)** Focal adhesion length was also measured and grouped into 3 groups (0-4, 4-8 and 8-12 μm). The graph shows % of the total FA per cell of the 3 length groups. Cells overexpressing ATS6 ASM, ATS6 FM and ATS10 WT had no significant increase in longer focal adhesions (4-8 μm). Statistical significance for deviation from the control values was calculated using 1-way ANOVA (2-way ANOVA for FA length comparisons) with a Bonferroni's multiple comparisons test using GraphPad Prism V6 (focal adhesion measurements per cell n>16). Asterisk indicate P values where * = P ≤ 0.05; ** = P ≤ 0.01. **(d)** EpH4 cells overexpressing ADAMTS6 (ATS6 WT) had significantly reduced pFAK in EpH4. Western blotting analysis for total FAK and pFAK is shown. Quantification of band intensity is shown as a ratio of the control band intensity. The western blot and quantitation shown is from a single representative experiment (n=3).

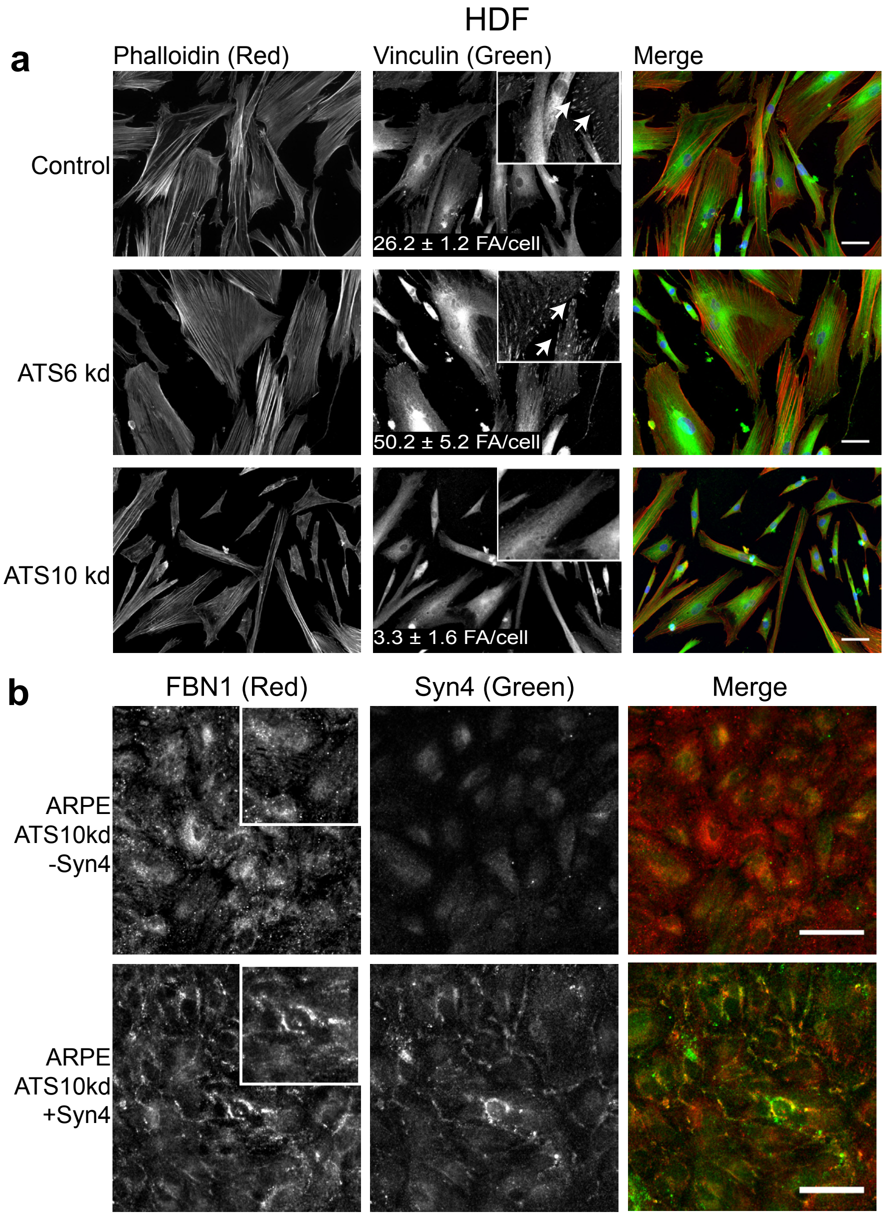

Supplementary Figure 2. Effects of ADAMTS knock-in and knock-down on focal adhesions.
(a) Immunofluorescence microscopy of human dermal fibroblasts (HDF) with siRNA treatment of ADAMTS10 (ATS10 kd) or ADAMTS6 (ATS6 kd). ATS6 kd cells had many more adhesions than control cells; ATS10 kd cells had no focal adhesions (n>5).
(b) Immunofluorescence microscopy of ARPE-19A cells with siRNA treatment of ADAMTS10 (ATS10 kd). Syndecan-4 (Syn4) over-expression rescued fibrillin-1 deposition, which co-localized along cell-cell boundaries with the increased syndecan-4. Scale bar indicates 50 μm.

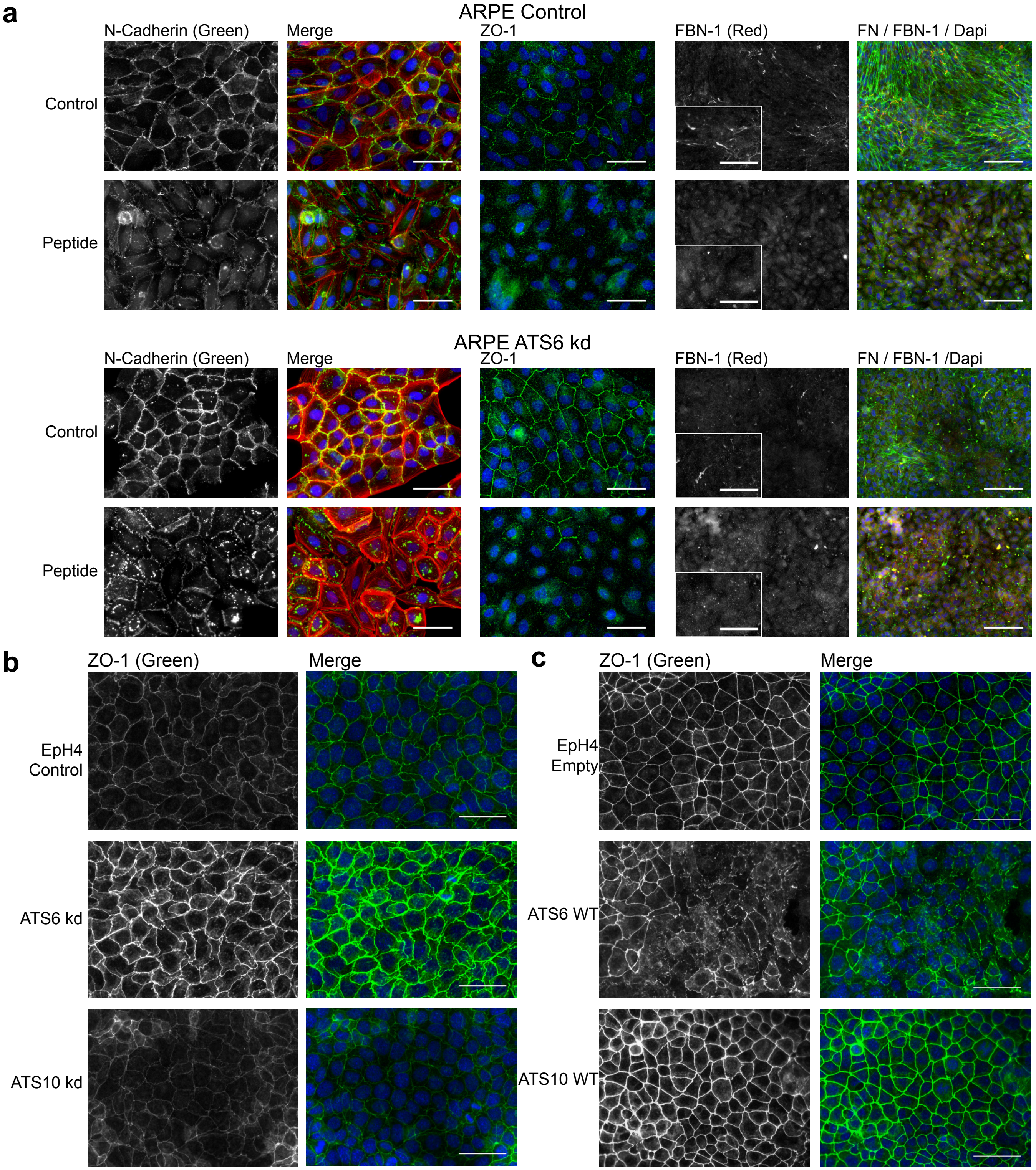

Supplementary Figure 3. ADAMTS6 and ADAMTS10 differentially alter cell junctions and epithelial fibrillin-1 microfibrils.

(a) Immunofluorescence microscopy of ARPE-19A cultured on glass coverslips for 3 days. Cells were treated with siRNA to down-regulate ADAMTS6 (ATS6 kd) and after 2 days cells were treated with overnight addition of anti-cadherin peptide (1mM). After fixation, cells were stained for phalloidin (B/W, red), N-cadherin (B/W) and ZO-1 (B/W, green), and nuclei were visualized with DAPI (blue). Images were taken with a 40x objective. Scale bar indicates 50 μm (inset 25 μm). Both fibrillin-1 (FBN1) and fibronectin (FN) deposition was disrupted on addition of cadherin peptide, as previously shown [1]. **(b)** and **(c)** Immunofluorescence microscopy of EpH4 cells cultured on glass coverslips for 7 days. Cells were treated with siRNAs to down-regulate either ADAMTS6 (ATS6 kd) or (ATS10 kd) (B)**,** or with lentivectors to overexpress ADAMTS6 (ATS6 WT) or ADAMTS10 (ATS10 WT) (C). ADAMTS10 supported tight junctions, but ADAMTS6 disrupted them. After fixation, cells were stained for ZO-1 (B/W, green), and nuclei were visualized with DAPI (blue). Images were taken with a 40x objective. Scale bars indicate 50 μm.

1. Baldwin, A. K., Cain, S. A., Lennon, R., Godwin, A., Merry, C. L. and Kielty, C. M. (2014) Epithelial-mesenchymal status influences how cells deposit fibrillin microfibrils. J. Cell Sci. 127, 158-171

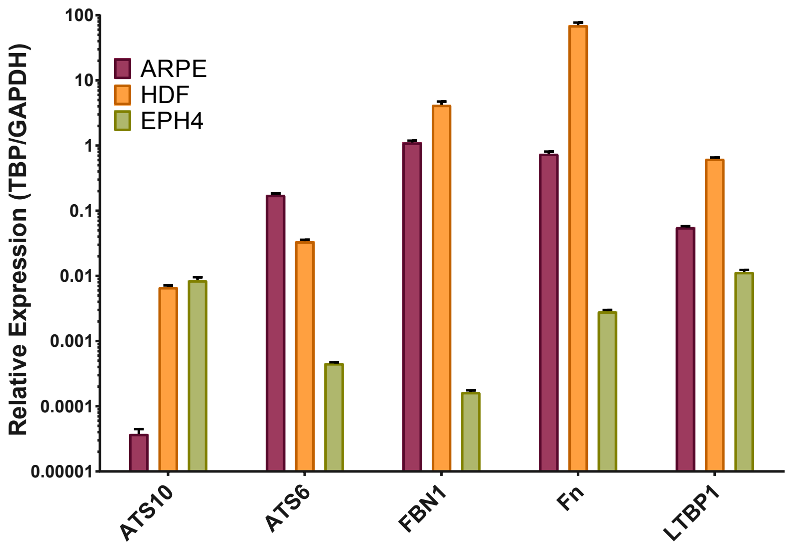

Supplementary Figure 4. Relative expression of ADAMTS10, ADAMTS6, fibrillin-1 and associated molecules, by epithelial and mesenchymal cells.

We determined the relative expression levels of ADAMTS10 (ATS10), ADAMTS6 (ATS6) and microfibrillar proteins, by reverse transcription quantitative real-time PCR (RT-qPCR), in ARPE-19A, EpH4 epithelial cells and adult HDFs. ARPE-19A cells expressed very low levels of ADAMTS10, over 4500-fold less than ADAMTS6. In contrast, HDFs expressed 5-fold-less ADAMTS10 than ADAMTS6 and EpH4 cells expressed 18-fold more ADAMTS10 than ADAMTS6. ARPE-19A cells expressed 4-fold less fibrillin-1 (FBN1) than HDFs, 100-fold less FN than HDFs, and 12-fold less LTBP-1 (latent TGFβ-binding protein which co-localizes with microfibrils [1]) than HDFs. EpH4 cells expressed less fibrillin-1 and FN than the other cell types.

1 Massam-Wu, T. et al. Assembly of fibrillin microfibrils governs extracellular deposition of latent TGFβ. J. Cell Sci. 123, 3006-3018, (2010).

Gene	Forward	Reverse
ADAMTS6	TACCATGGCCGCAAAGACAT	TCCTAGGCTGGAATCACGGT
FBN1	GGGCATTTGCCAGAACAC	CGCTGAGGCATTCGTTTT
Fn	CTGCGAGAGCAAACCTGAAG	TTTAGGACGCTCATAAGTGTCAC
LTBP1	ACCAAGGGCTTCCTGTCC	CGGGGTAGACGTGAGGAA
GAPDH	CCGCATCTTCTTTTGCGTCG	ACCAAATCCGTTGACTCCGA
TBP	GTGACCCAGCATCACTGTTTC	GAGCATCTCCAGCACACTCT

Supplementary Table 1. qPCR primer sequences.
